# nosoi: A stochastic agent‐based transmission chain simulation framework in r

**DOI:** 10.1111/2041-210X.13422

**Published:** 2020-06-21

**Authors:** Sebastian Lequime, Paul Bastide, Simon Dellicour, Philippe Lemey, Guy Baele

**Affiliations:** ^1^ Department of Microbiology, Immunology and Transplantation Rega Institute KU Leuven Leuven Belgium; ^2^ Cluster of Microbial Ecology Groningen Institute for Evolutionary Life Sciences University of Groningen Groningen The Netherlands; ^3^ IMAG CNRS University of Montpellier Montpellier France; ^4^ Spatial Epidemiology Lab (SpELL) Université Libre de Bruxelles Brussels Belgium

**Keywords:** agent‐based simulation, infectious disease, pathogen, r package, simulator, stochastic model, transmission chain

## Abstract

1. The transmission process of an infectious agent creates a connected chain of hosts linked by transmission events, known as a transmission chain. Reconstructing transmission chains remains a challenging endeavour, except in rare cases characterized by intense surveillance and epidemiological inquiry. Inference frameworks attempt to estimate or approximate these transmission chains but the accuracy and validity of such methods generally lack formal assessment on datasets for which the actual transmission chain was observed.
2. We here introduce nosoi, an open‐source r package that offers a complete, tunable and expandable agent‐based framework to simulate transmission chains under a wide range of epidemiological scenarios for single‐host and dual‐host epidemics. nosoi is accessible through GitHub and CRAN, and is accompanied by extensive documentation, providing help and practical examples to assist users in setting up their own simulations.
3. Once infected, each host or agent can undergo a series of events during each time step, such as moving (between locations) or transmitting the infection, all of these being driven by user‐specified rules or data, such as travel patterns between locations.
4. nosoi is able to generate a multitude of epidemic scenarios, that can—for example—be used to validate a wide range of reconstruction methods, including epidemic modelling and phylodynamic analyses. nosoi also offers a comprehensive framework to leverage empirically acquired data, allowing the user to explore how variations in parameters can affect epidemic potential. Aside from research questions, nosoi can provide lecturers with a complete teaching tool to offer students a hands‐on exploration of the dynamics of epidemiological processes and the factors that impact it. Because the package does not rely on mathematical formalism but uses a more intuitive algorithmic approach, even extensive changes of the entire model can be easily and quickly implemented.

## Résumé

1. Le processus de transmission d'un agent infectieux crée une chaîne connectée d'hôtes, connue sous le nom de chaîne de transmission. Reconstruire cette dernière reste une entreprise difficile, sauf dans de rares cas caractérisés par une surveillance épidémiologique intense. Il existe des méthodes d'inférence pour estimer ou approximer ces chaînes de transmission. Cependant la précision et la validité de ces méthodes ne sont généralement pas formellement évaluées sur des jeux de données pour lesquelles la chaîne de transmission est connue.
2. Nous présentons ici nosoi, un progiciel r libre qui propose une infrastructure individu‐centrée complète, réglable et extensible, pour simuler des chaînes de transmission dans une grande variété de scénarios épidémiologiques. nosoi est disponible sur GitHub et le CRAN, et est accompagné d'une documentation étendue munie d'exemples pratiques détaillés permettant aux utilisateurs de paramétrer aisément leurs propres scénarios de simulation.
3. Une fois infecté, chaque hôte ou agent peut subir une série d'événements à chaque pas de temps, tel que bouger (entre deux lieux) ou transmettre l'infection, chacun de ces événements étant spécifié par des règles ou des données fournies par l'utilisateur.
4. nosoi est capable de générer une multitude de scénarios épidémiques, pouvant, par exemple, être utilisés pour valider certaines méthodes de reconstruction, dont les modélisations épidémiologiques et les analyses phylodynamiques. nosoi offre également une infrastructure complète pour tirer parti de données acquises empiriquement, permettant l'exploration de l'influence des divers paramètres d'intérêt sur le potentiel épidémique. En plus de son utilisation en recherche, nosoi procure aux enseignants universitaires un outil complet pour explorer avec leurs étudiants la dynamique des processus épidémiologiques et ses paramètres. Ce progiciel ne reposant pas sur un formalisme mathématique mais utilisant une approche algorithmique plus intuitive, des changements même drastiques de l'entièreté du modèle peuvent être facilement et rapidement implémentés.

## 1 INTRODUCTION

Infectious disease events, especially those resulting from novel emerging pathogens, have significantly increased over the past few decades, possibly as a result of alterations in various environmental, biological, socioeconomic and political factors (Chan et al., 2010). By definition, infectious agents need to spread through transmission between hosts. If successful, the resulting transmission process creates a connected chain of hosts linked by transmission events, usually called a transmission chain. Transmission is highly stochastic and can be influenced by a wide array of intrinsic and extrinsic factors, such as within‐host dynamics and environmental or host behavioural factors. Reconstruction of transmission chains, however, remains difficult to achieve, except in certain rare cases characterized by intense surveillance and epidemiological inquiry (Mollentze et al., 2014; Worby et al., 2016).

Molecular data may represent a critical asset in reconstructing the transmission history of a pathogen (Campbell, Cori, Ferguson, & Jombart, 2019; De Maio, Worby, Wilson, & Stoesser, 2018; Didelot, Fraser, Gardy, & Colijn, 2017; Didelot, Gardy, & Colijn, 2014; Worby et al., 2016). Often, however, the relationship between individual cases is too distant to allow for the perfect reconstruction of a transmission chain. In that context, the study of infectious agents' genomic sequences can be used to reconstruct, under an evolutionary model, their likely evolutionary history. These reconstructions rely on evolution occurring on the same time‐scale as the epidemic or transmission process, which is the case for most fast‐evolving pathogens such as RNA viruses (Romero‐Severson, Skar, Bulla, Albert, & Leitner, 2014; Ypma, van Ballegooijen, & Wallinga, 2013). The inferred evolutionary history has been used in recent years to estimate the timing, the origin or the effectiveness of mitigation measures of several epidemics (Dellicour et al., 2018; Dudas, Carvalho, Rambaut, & Bedford, 2018; Grubaugh et al., 2019; Hill et al., 2019).

The accuracy, validity or limitations of both currently available and future methods, however, generally lack formal assessment on datasets for which we have been able to observe the actual geographical spread and the complex factors that shaped its pattern. In that context, a simulated dataset is extremely useful as the exact transmission history is known and can be compared to the histories inferred from different software packages. The last decade has seen the development of several integrated epidemic and genetic simulation tools that can be used to assess the performance of some of these models, such as treesim (Stadler & Bonhoeffer, 2013), seedy (Worby & Read, 2015), outbreaker2 (Campbell et al., 2018) or favites (Moshiri, Ragonnet‐Cronin, Wertheim, & Mirarab, 2019).

While undoubtedly useful, these tools fall short in accommodating a wide range of epidemiological scenarios. In particular, arboviral (e.g. Zika, dengue or yellow fever) outbreaks, where two types of hosts participate in the epidemic process, are poorly modelled. These hosts are characterized by drastically different behaviour or infection dynamics and cannot be accurately modelled using a single host type. Furthermore, geographical location diffusion is simulated in these tools, when possible, on a contact network or in discrete space. Yet, recent years have seen the development of methods taking advantage of phylogeographical diffusion in continuous space (Dellicour, Rose, Faria, Lemey, & Pybus, 2016; Lemey, Rambaut, Welch, & Suchard, 2010), creating a need for epidemiological simulations in a continuous space.

To enable the performance assessment of these methods under complex and realistic scenarios, including spread in continuous space or arbovirus outbreaks, we present nosoi, a flexible agent‐based transmission chain simulator implemented as an open‐source r package (R Core Team, 2019).

## 2 CHARACTERISTICS

**Figure 1:**
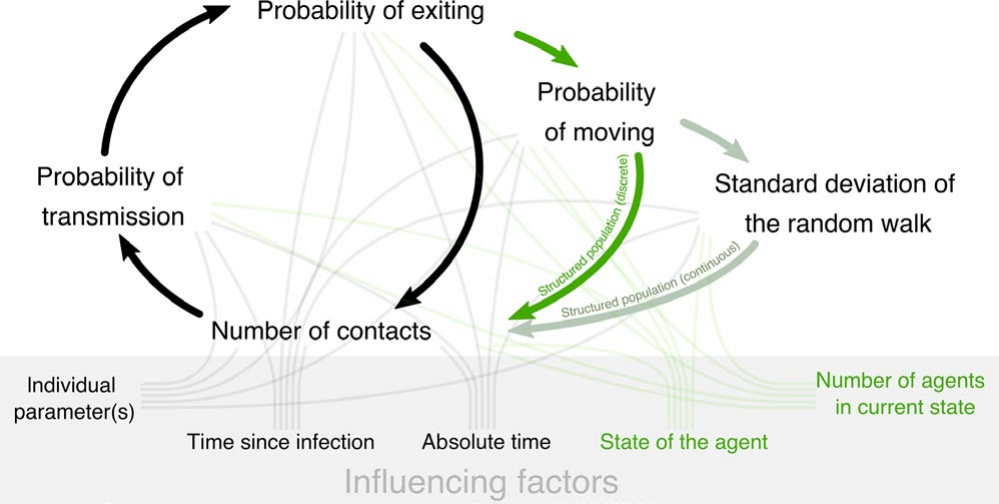
Schematic of status and location assessment for each agent (in case of a structured population), or host, during each discretized time step of the simulation. Optional steps in the simulation framework are shown in shades of green and are only performed in case of a structured (either discrete or continuous) population. Several factors (embedded in the gray box), either individually or globally set, can influence these steps according to user‐specified settings

nosoi generalizes and significantly extends a basic model that allowed individual humans and mosquitoes—each one being characterized by a unique set of infection parameters—to interact within a simulated environment (Fontaine et al., 2018). It was initially designed to model real‐world arboviral epidemics unfolding under varying within‐host dynamics (Fontaine et al., 2018).

nosoi employs agent‐based modelling, which focuses on the individual active entities—known as (autonomous) agents—of a system and defines their behaviour and the interactions between them. The main interest then lies in the global dynamics of and the complex phenomena within the system that emerges from the interactions of the many individual behaviours. Within nosoi, the agents' behaviour is governed by user‐specified rules that can accommodate high levels of stochasticity at each level of the epidemic process. Agents can experience dual‐host dynamics, such as those from human and mosquito populations, and exist in structured populations, with different behaviours according to host type and/or structure. Population structure can either be absent, discrete (e.g. different categories) or continuous (such as geographical space). In these structures, agents can trigger a movement, a contact or a transmission event, with the probability of such an event occurring being potentially host‐, individual‐, structure‐ and/or time‐dependent. These agents are recruited when infected and can either recover or die from the infection, resulting in their removal from the simulation. The status and location of each agent are assessed according to the model during each step of the discretized time of the simulation (Figure 1). The simulation ends when the user‐specified value of the number of infected agents or when the targeted simulation time is reached.

In essence, nosoi allows the user to simulate and keep track of one or more transmission chains occurring during an infectious disease outbreak and, as such, to store and output a (collection of) transmission tree(s). Genetic data can be subsequently simulated along each transmission tree using sequence simulation software such as πbuss (Bielejec et al., 2014) or SantaSim (Jariani et al., 2019), which can then serve as input for phylodynamic inference methods. nosoi is accompanied by extensive tutorials, helping the user to set up and visualize their simulation, available as documentation in the package, or at https://slequime.github.io/nosoi /.

## 3 PRACTICAL EXAMPLE

We here showcase nosoi with the starting scenario of a single human infected with an Ebolavirus‐like pathogen in West Africa. The simulated epidemic unfolds in a geographically structured host population, specifically in a continuous geographic space, for 365 days or discrete time‐steps. Within‐host dynamics, influencing the probability of exiting the simulation (dying or recovering) and the between‐host transmission probability, are modelled according to published literature that describes Ebolavirus infection in humans (Casillas, Nyamathi, Sosa, Wilder, & Sands, 2003; Skrip et al., 2017). The remaining parameters (number of daily contacts, probability of movement and standard deviation of the random walk in continuous space) were empirically set. The number of daily contacts is restricted by the number of people living in the area, as provided by spatial demographics data obtained from WorldPop (www.worldpop.org), to avoid reaching locally unrealistic counts of infected humans. The complete specification and accompanying code for this simulation are available as a document on nosoi's website (https://slequime.github.io/nosoi/articles/examples/ebola.html).

Over the course of 365 days, the simulation has yielded 3,603 infected agents. The average number of secondary cases per agent is 1.12, which is roughly coherent with previous epidemiological estimates of *R*_0_ for previous Ebolavirus outbreaks (Van Kerkhove, Bento, Mills, Ferguson, & Donnelly, 2015). The increase in infected agents' number is exponential, as would be expected considering the specifications of the model, that is, absence of intervention strategies or changes in the simulated environment.

**Figure 2:**
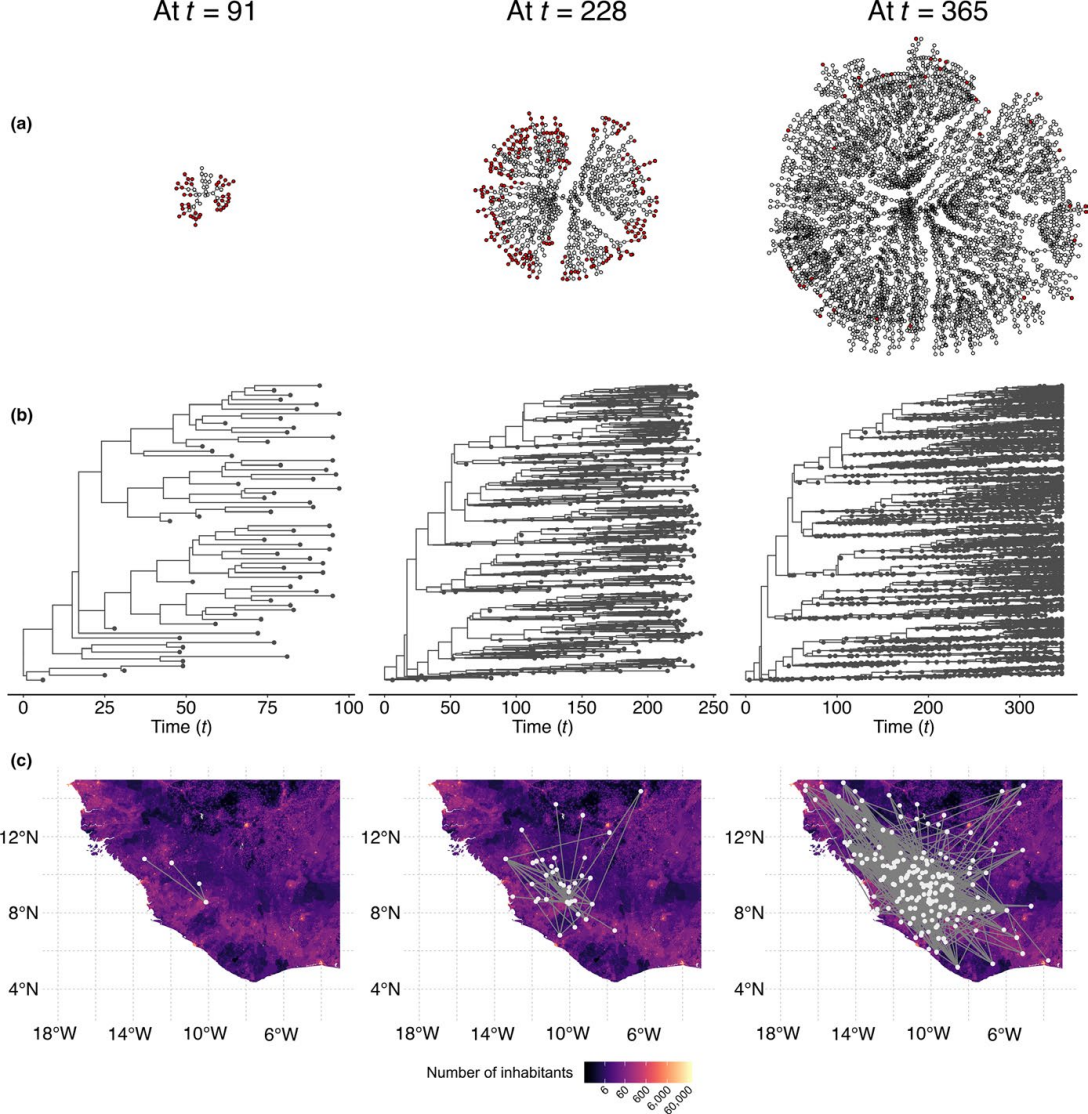
Visualization of a simulated Ebolavirus‐like transmission chain in West Africa at three time‐points (91, 228 and 365 days after the introduction of the first infected host), represented as (a) a network, (b) a tree or (c) a tree mapped on the continuous space the simulation took place in

The transmission chain can be represented either as a network (Figure 2a) or as a tree (Figure 2b) that can be mapped in the continuous space in which the epidemic took place (Figure 2c). The tree representation of the transmission chain can be seen as the genealogy of the pathogen population over which molecular evolution generates the observed sequence data, then used to reconstruct this same history. In this representation, each internal node is a transmission event, each tip represents the exit point in time of an agent, and the root is the starting point in time of the initially infected agent. Branches or sets of connected branches represent the life span of each agent. This tree is binary, counts as many tips as the total number of infected agents and as many internal nodes as transmission events.

Other examples are available on nosoi's website illustrating various scenarios, such as spread of a dengue‐like pathogen (dual‐host) in a discrete space or an unstructured population of hosts. The tutorials also provide guidelines on how to set up simulations in various combinations of settings currently available.

## 4 USES

Trends in globalization, including expansion in international travel and trade, have extended the reach and increased the pace at which infectious diseases spread (Chan et al., 2010). These trends provide infectious agents with ample opportunities to establish and spread successfully, but many practical difficulties remain in accurately inferring key aspects of an epidemic. Standard testing of models of spread typically focuses on using that same model to generate simulated data, which offers important but limited insights and mostly provides a test of proper implementation and a way to compare different methodologies. nosoi, however, is a phylogenetic model‐independent agent‐based simulation framework that offers realistic and complex epidemiological scenarios. As such, it enables accurate testing of popular inference methods in both discrete and continuous phylogeography using either maximum‐likelihood (Ishikawa, Zhukova, Iwasaki, & Gascuel, 2019) or Bayesian inference (Lemey, Rambaut, Drummond, & Suchard, 2009; Lemey et al., 2010; Suchard et al., 2018), which are widely used in pathogen phylodynamics. In that regard, an interesting application of our proposed simulation framework could be to study the increasingly popular structured coalescent models (Bouckaert et al., 2019; De Maio, Wu, O'Reilly, & Wilson, 2015; Müller, Rasmussen, & Stadler, 2017), and to compare their accuracy under realistic epidemiological transmission scenarios against discrete phylogeographical inference.

nosoi enables the simulation of real‐life scenarios of viral outbreaks, and we provide several example scenarios to showcase its capabilities to generate a single transmission chain using different settings. An important aspect is that the resulting transmission tree, which describes the transmission events between infected hosts, differs from the phylogenetic tree, which describes the ancestral genetic relationships between pathogens sampled from these hosts. In that regard, it is crucial to acknowledge the growing number of methods that infer either phylogenetic trees, transmission trees or jointly estimate both (for an overview, we refer to Baele, Suchard, Rambaut, and Lemey (2017)).

Apart from assessing the performance of various methods in reconstructing geographical spread or the dynamics of an infectious agent, nosoi can prove useful for assessing the performance of classic deterministic SIR and SIRS compartmental models (Kermack & McKendrick, 1927). These epidemiological models estimate the theoretical number of people infected with a contagious illness in a closed population over time under some assumptions. For example, the original SIR model assumes that the population size is fixed, that the incubation period of the infectious agent is instantaneous and that the duration of infectivity is the same as the length of the disease. It also assumes a completely homogeneous population with no age, spatial or social structure. These assumptions can be matched as closely as possible by the user‐defined settings in nosoi or be violated in more realistic settings, allowing to examine the sensitivity of the deterministic models to the assumptions under a complex and fine‐tuned epidemiological scenario.

nosoi also offers, in line with its initial purpose (Fontaine et al., 2018), a comprehensive framework to leverage empirically acquired data. A pathogen's within‐host dynamics characterized in laboratory settings can be embedded into a full stochastic epidemiological model, allowing the user to explore how variation can affect its epidemic potential.

Aside from research questions, nosoi can provide lecturers with a complete teaching tool to offer students a hands‐on exploration of the dynamics of epidemiological processes and the factors that impact it. Because the package does not rely on mathematical formalism but uses a more intuitive algorithmic approach, even extensive changes of the entire model or part of it can be easily and quickly implemented. The documentation provides suggestions for visualization using well‐known external r‐packages, such as ggplot2 (Wickham, 2009) or ggtree (Yu, Lam, Zhu, & Guan, 2018; Yu, Smith, Zhu, Guan, & Lam, 2016). The package is also fully integrated in the r and phylogenetic environments, and, through the use of the treeio and tidytree r packages (Wang et al., 2019), simulated transmission trees can be exported in a wide variety of formats for downstream analyses, such as the beast (Suchard et al., 2018) or jplace (Matsen, Hoffman, Gallagher, & Stamatakis, 2012) formats.

In summary, nosoi provides a complete, tunable and expandable framework to simulate epidemiological processes based on transmission chains, in a user‐friendly manner. Accessible through GitHub and the CRAN, the code is well covered by unitary tests and accompanied by extensive documentation, providing help and practical examples to users. Open‐source and coded in the widely used r language, it allows users to customize their model by implementing new mechanisms for all or part of the core model. In addition, and contrary to other available tools, by decoupling sequence evolution from the epidemiological process, it can connect to any external sequence simulator, allowing the user to choose a tool and model that can address the biological question of interest.

## AUTHORS' CONTRIBUTIONS

S.L. designed and conceived the package, and wrote its documentation; P.B. and S.D. provided editing and optimization to the package r code; P.L. and G.B. supervised and guided the project; S.L. and G.B. wrote the initial draft. All authors contributed critically to the drafts and gave final approval for publication.

## Data Availability

The package is available on GitHub (https://github.com/slequime/nosoi) and the CRAN (https://cran.r‐project.org/package=nosoi). The reviewed version of the package presented in this manuscript is available through Zenodo (https://doi.org/:10.5281/zenodo.3860006). The complete specification and accompanying code for the simulation presented in this manuscript are available as a document on nosoi's website (https://slequime.github.io/nosoi/articles/examples/ebola.html).
